# The association between Urban and Rural Resident Basic Medical Insurance and instrumental activity of daily living disability among middle-aged and older adults in China

**DOI:** 10.1186/s13690-023-01167-3

**Published:** 2023-08-31

**Authors:** Jian Sun, Shui Yu, Wanjun Lu, Yujiang Liu

**Affiliations:** 1 College of Public Administration, Nanjing Agricultural University, 1 Weigang, Xuanwu District, Nanjing, 210095 China; 2 Research Center for Local Governance and Policy, Nanjing Agricultural University, 1 Weigang, Xuanwu District, Nanjing, 210095 China; 3 China Resources Environment and Development Academy, Nanjing Agricultural University, 1 Weigang, Xuanwu District, Nanjing, 210095 China; 4 Jin Shanbao Institute for Agriculture & Rural Development, Nanjing Agricultural University, 1 Weigang, Xuanwu District, Nanjing, 210095 China

**Keywords:** Urban and Rural Resident Basic Medical Insurance, Instrumental activity of daily living disability, Middle-aged and older adults, China Health and Retirement Longitudinal Study, China

## Abstract

**Background:**

Previous studies have not investigated the association between medical insurance and instrumental activity of daily living (IADL) disability. To fulfill this research gap, this study aims to explore the association between Urban and Rural Resident Basic Medical Insurance (URRBMI) and IADL disability among middle-aged and older adults in China.

**Methods:**

The data of this study were sourced from the 2018 wave of China Health and Retirement Longitudinal Study (CHARLS). Logit regression models were used to analyze the association between URRBMI and odds of suffering from IADL disability. Furthermore, we used IV-Probit regression model to address the potential endogeneity problem. Moreover, propensity score matching and generalized random forest model were employed to conduct robustness checks.

**Results:**

The logit regression results reveal that URRBMI participation was significantly related to reduced odds of suffering from IADL disability by 39.86% after adjusting for the control variables (*p* < 0.01). The results of IV-Probit estimation show that URRBMI was an exogenous variable. Further robustness checks reported similar estimation results. The results of heterogeneity analysis reveal that URRBMI produced a statistically stronger effect on IADL disability for the older adults (OR = 0.5815, *p* < 0.01) when compared with the middle-aged adults (OR = 0.5690, *p* < 0.05). The results of impact channel analysis indicate that physical exercise was a channel involving the effect of URRBMI on IADL disability.

**Conclusion:**

This study finds that the middle-aged and older adults who were covered by URRBMI had a reduced possibility of suffering from IADL disability when compared with those without URRBMI. Furthermore, it is found that URRBMI produced a statistically stronger effect on IADL disability for the older adults when compared with the middle-aged adults. Moreover, we obtain evidence indicating that physical exercise was a channel involving the effect of URRBMI on IADL disability.

**Table:** 

**Text box 1. Contributions to the literature**
• Previous studies ignored the association between medical insurance and IADL disability. This study finds that the middle-aged and older adults who were covered by URRBMI had a reduced possibility of suffering from IADL disability when compared with those without URRBMI.
• The heterogeneity analysis reveals that URRBMI produced a statistically stronger effect on IADL disability for the older adults when compared with the middle-aged adults.
• The impact channel analysis reveals that physical exercise was a channel involving the effect of URRBMI on IADL disability.
• From the perspective of methods utilization, this study used the generalized random forest model from machine learning to conduct a robustness check, which encourages the use of more machine learning methods in health issue.

## Background

World Health Organization (WHO) defines health as a state of complete physical, mental, and social well-being, not merely the absence of disease or infirmity [1]. That is to say, physical health is an important part of health. Generally speaking, people who aged 45 years and older will show presenile characteristics, such as slow metabolism, decreased resistance, and decreased physiological functions [2], and then enter the potential stage of aging [3]. Deaton pointed out that the focus of human health has shifted from infants to middle-aged and older adults [4].

Functional disability refers to diminished capacity or inability to perform basic self-care tasks that are usually required for independent living in the community [5]. Functional ability deteriorates progressively as people age [6]. The ability to function independently in the community is a critically important public health issue [7]. Functional disability prevents the middle-aged and older adults from performing some social roles, affects quality of life and well-being, and even improves suicide risk [8]. In addition, functional disability places a large burden on health services [9]. Previous studies reveal that higher socioeconomic status is correlated with a lower incidence rate of disability [10–12]. Both activities of daily living (ADL) disability and instrumental activities of daily living (IADL) disability belong to functional disability. The former refers to diminished capacity or inability to complete activities of daily living, while the latter refers to diminished capacity or inability to use instruments to complete activities of daily living.

In 2017, the National Health and Family Planning Commission and other 12 departments in China issued the National 13th Five-Year Plan for Healthy Aging, which stated that about 40.63 million Chinese older adults were in disability or partial disability in 2015. Furthermore, with the accelerated urbanization, more and more Chinese young people migrate to cities for work, while their parents are left in rural areas. Moreover, accompanying the trend of family miniaturization, it is estimated that the number of empty-nest older adults will be unprecedentedly large in the next 30 years [13]. In 2020, the outbreak of Corona Virus Disease 2019 (COVID-19) has posed daunting challenges to people's health outcome and daily lives. It is important to note that the middle-aged and older adults are one of the most susceptible populations to COVID-19 [14].

In 1990, WHO put forward the concept of healthy aging. The core notion of healthy aging is to improve the health outcome of the older adults, which has become an important basis for several countries to formulate health policies for the older adults. Furthermore, the National 13th Five-Year Plan for Healthy Aging of China noted that comprehensive and systematic interventions should be carried out on all the factors that affect health outcome of the older adults from the early stage of life to create a social supporting and living environment, so as to prolong healthy life expectancy, maintain health function, and improve health outcome.

In 2016, the Chinese government merged Urban Residents Basic Medical Insurance (URBMI) and New Rural Cooperative Medical Scheme (NRCMS) to form Urban and Rural Resident Basic Medical Insurance (URRBMI). As an important social policy, URRBMI integrates urban and rural residents into the same medical security system, which marks the improvement of medical security equity. URRBMI has higher reimbursement rates, which is useful to reduce economic burden of diseases. URRBMI can be used in more medical institutions, which significantly improves spatial accessibility of health services. Furthermore, the purpose of URRBMI is to realize the mutual aid of urban and rural residents in terms of economic burden of disease, narrow the health inequality between urban and rural residents, and improve their health outcome.

Theory of demand for health was put forward by Grossman [15], which holds that individual factors, such as age, education, and income, can affect health outcome. The theory of demand for health can be used to explain individual health outcome and its influencing factors. Extended model of theory of demand for health was proposed by Leibowitz [16], which holds that both individual and environmental factors can affect health outcome, and it is necessary to include both individual and environmental factors into the health production function. In this study, we incorporated URRBMI into the theory of demand for health as a factor affecting health outcome. In addition, the extended model of theory of demand for health provides references for control variables selection of this study.

Previous studies have explored the association between medical insurance and functional disability. For example, Cheng et al. used the 2005 and 2008 waves of Chinese Longitudinal Healthy Longevity Survey (CLHLS) and found that participation of NRCMS significantly reduced the possibility of suffering from ADL disability among the older adults in China [17]. Zhang et al. used the 2011 and 2013 waves of China Health and Retirement Longitudinal Study and confirmed the positive effect of NRCMS on ADL disability [18]. Health services utilization refers to a general term for health services utilized by individuals to maintain or improve health outcome. Physical exercise refers to a health behavior which is useful to maintain or improve health outcome. Some studies reveal that medical insurance can promote health services utilization and physical exercise [19–22]. Furthermore, health services utilization and physical exercise are beneficial to improving health outcome [23–28]. Consequently, we hypothesized that URRBMI may affect IADL disability by promoting health services utilization and physical exercise.

Overall, we find that previous studies concerning the association between medical insurance and functional disability are quite limited. Specifically speaking, previous studies mainly focused on exploring the relationship between medical insurance and ADL disability, and ignored the association between medical insurance and IADL disability. IADL disability is a relatively objective health indicator when compared with ADL disability. IADL disability has been widely used to measure physiological health [29, 30], and it is of great significance to analyze the association between medical insurance and IADL disability. To fulfill this research gap, this study aims to explore the association between URRBMI and IADL disability among the middle-aged and older adults in China. Furthermore, we explored whether the association between URRBMI and IADL disability differs by age and residency area. Last but not least, we investigated the impact channels for the association between URRBMI and IADL disability among the middle-aged and older adults.

## Methods

### Data source

**Fig. 1:**
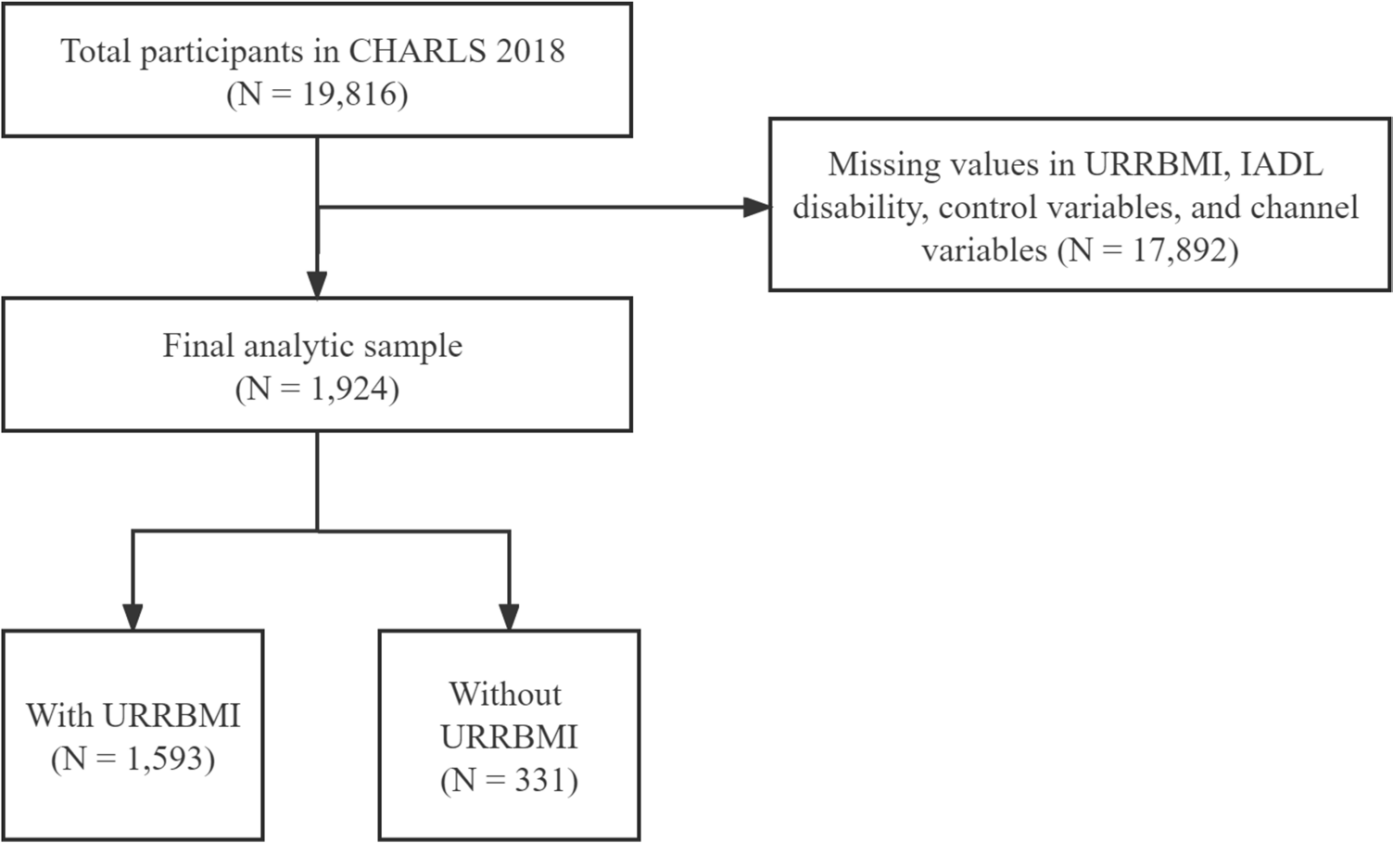
Sample selection procedure of this study CHARLS indicates China Health and Retirement Longitudinal Study. URRBMI indicates Urban and Rural Resident Basic Medical Insurance. IADL indicates instrumental activity of daily living

The data of this study were sourced from the 2018 wave of China Health and Retirement Longitudinal Study (CHARLS). It is important to notice that we have tried to use 4 waves of CHARLS, whilst the sample size decreased below 500, which is difficult to reflect the policy effectiveness of URRBMI. Consequently, we decided to use the latest wave of CHARLS. Given the fact that the CHARLS contains rich information about the middle-aged and older adults that we need, we decided to use it investigate the association between URRBMI and IADL disability among the middle-aged and older adults in China. In addition, it is important to notice that the data of Particulate Matter 2.5 (PM_2.5_) concentrations used in this study were obtained from Atmospheric Composition Analysis Group. After removing the observations with missing values, this study included 1,924 participants who aged 45 years and older (Fig. 1).

### Variables

#### Dependent variable

In this study, IADL disability is the dependent variable, which was measured by IADL scale. The IADL scale reflects the middle-aged and older adults’ abilities to use instruments to complete activities of daily living. Furthermore, this scale includes the middle-aged and older adults’ status of shopping, making phone calls, cooking, doing household chores, taking medications, and managing money. The response for each question was ‘No, I don’t have any difficulty’, ‘I have difficulty but can still do it’, ‘Yes, I have difficulty and need help’, or ‘I can not do it’. The participants were categorized as having IADL disability (coded as 1) if they had any difficulty in performing any of the above-mentioned six items (otherwise coded as 0). In this study, Cronbach’s α of the IADL scale was 0.775. This indicates that the scale has good internal consistency and meets the reliability requirement, which is consistent with Mirzadeh et al. [31], who also found that the reliability of the IADL scale was significantly high.

#### Independent variable

In this study, Urban and Rural Resident Basic Medical Insurance (URRBMI) is the independent variable. URRBMI is a dummy variable, which indicates whether the adult had URRBMI or not (having URRBMI coded as 1, not having URRBMI coded as 0).

#### Control variables

According to the extended model of theory of demand for health [16], we selected three types of control variables that were associated with health outcome. The first type of control variables described the demographic characteristics, which include age (continuous variable), gender (1 = male, 0 = female), marital status (1 = married, 0 = single, divorced or widowed), and residency area (1 = living in urban areas, 0 = living in rural areas). The second type of control variables described the socioeconomic status, including education status (1 = literate, 0 = illiterate) and household income (logarithm value). The third type of control variables described the residential environment, including PM_2.5_ concentrations (continuous variable) and having tap water (1 = yes, 0 = no).

Table 1 reports Variance Inflation Factor (VIF) test results, suggesting that all the VIF values of the variables that we employed were much lower than 10. Consequently, there was no serious multicollinearity.

**Table 1:** Results of Variance Inflation Factor test

Variable	VIF	1/VIF
Age	1.19	0.84
Education status	1.17	0.86
Marital status	1.15	0.87
Gender	1.10	0.91
Residency area	1.08	0.92
Having tap water	1.04	0.96
PM_2.5_ concentrations	1.04	0.96
Household income	1.03	0.97
URRBMI	1.03	0.97
Mean VIF	1.09	

VIF indicates Variance Inflation Factor. PM_2.5_ indicates Particulate Matter 2.5. URRBMI indicates Urban and Rural Resident Basic Medical Insurance

#### Channel variables

Two channel variables were selected in this study. The first channel variable described health services utilization (including outpatient and inpatient services utilization, 1 = yes, 0 = no). The second channel variable described physical exercise (1 = yes, 0 = no).

### Statistical analyses

Considering the fact that IADL disability was a dummy variable, logit regression models were used to analyze the association between URRBMI and odds of suffering from IADL disability among the middle-aged and older adults in China. It is important to notice that we reported robust standard errors that clustered at the community level to reduce the potential effect of heteroscedasticity. The logit regression model for the association between URRBMI and odds of suffering from IADL disability can be written as follows:

1 $$Logit\left(Y\right)=Ln\frac{{P}_{i}}{1-{P}_{i}}={\alpha }_{0}+{\alpha }_{1}*{URRBMI}_{i}+{\sum }_{j=1}^{n}{\alpha }_{j}*{X}_{ji}+{\varepsilon }_{i}$$

where *P*_*i*_ indicates the possibility of suffering from IADL disability for adult *i*, *P*_*i*_ / (1—*P*_*i*_) represents the odds of suffering from IADL disability, *URRBMI*_*i*_ denotes whether the adult was covered by URRBMI, *X*_*ji*_ suggests the control variables, *α*_0_ is the intercept term, *α*_1_ stands for the coefficient of URRBMI, which is our main interest, *α*_*j*_ indicates coefficients of the control variables, and *ε*_*i*_ is the error term.

Some unobservable factors, such as personality, may be correlated with participation of URRBMI and IADL disability at the same time. In addition, the middle-aged and older adults with IADL disability may be more likely to participate in URRBMI. Consequently, endogeneity problem may exist in this study. Instrumental variable (IV) method is an important method to address the potential endogeneity problem which is caused by reverse causation and omitted variable bias [32]. Given the fact that the dependent variable used in this study is a dummy variable, we used IV-Probit regression model to address the potential endogeneity problem. In this study, we used participation rate of URRBMI at the community level as an IV. The participation rate of medical insurance at the community level is significantly correlated with the participation of medical insurance [33]. In addition, the participation rate of URRBMI at the community level is an exogenous variable and does not produce a direct effect on IADL disability.

Considering the fact that URRBMI is a voluntary plan, the middle-aged and older adults can choose whether to participate in it or not. Therefore, sample selection bias may exist in this study. Given the fact that propensity score matching (PSM), which was originally developed by Rosenbaum and Rubin [34], can address the sample selection bias, make observational data close to random trial data, and obtain robust estimation results [35–38], we used it to conduct a robustness check.

Generalized random forest model, which was originally developed from random forest model [39], can be regarded as an application of random forest model in causal inference. The goal of the generalized random forest model is to maximize the variations of treatment effects between different tree model nodes [40]. In addition, this model can be applied to the estimation of any local moment condition and has advantages in causal inference [41]. In this study, the generalized random forest model was employed to conduct a robustness check. Liu et al. found that the number of decision trees in random forest model is crucial to the estimation results [42]. When the number of decision trees is small, the classification error of random forest model is large and the performance of estimation is poor. Following Liu et al. [43], 500, 1000, and 2000 decision trees were employed to compare the estimation results of the generalized random forest models, respectively.

Following Cutler and Lleras-Muney [44], we used the impact channel analysis method to investigate how URRBMI affects IADL disability among the middle-aged and older adults in China. The basic idea of impact channel analysis is that if URRBMI influences IADL disability through health services utilization and physical exercise, the absolute value of coefficient of URRBMI will become smaller, or the effect of URRBMI on IADL disability will change from statistically significant to insignificant when the channel variables are controlled in the regression models. This method has been widely used in previous studies [45–49]. In order to facilitate the comparison of coefficient changes, linear probability regression models were employed for impact channel analysis in this study. The regression model can be written as follows:

2 $$IADL\;{disability}_i=\beta_0+\beta_1\ast{URRMI}_i+\sum\nolimits_{j=1}^n\beta_j\ast X_{ji}+\beta_2\ast C_i+\varepsilon_{it}$$

where *C*_*i*_ denotes the channel variables, *β*_0_ indicates the intercept term, *β*_1_,* β*_*j*_, and* β*_2_ stands for the coefficients of URRBMI, control variables, and channel variables, respectively. Moreover, 1-*β*_1_/*α*_1_ indicates the relative importance of each channel in explaining the relationship between URRBMI and IADL disability.

## Results

### Descriptive statistics

Table 2 presents the descriptive statistics of all the variables used in this study. Approximately 83% of the participants were insured by URRBMI, and about 22% of them suffered from IADL disability. Furthermore, about 36% of the participants aged 65 years and older, and more than 52% of them were female. More than 86% of the participants were married, and about 38% of them lived in urban areas. In addition, more than 80% of the participants were illiterate, and the mean value of household income was RMB 27,789.56 (US$ 4,271.26). PM_2.5_ concentrations has a mean value of 34.37 and a standard deviation of 14.65, and about 83% of the participants had tap water to use. Approximately 26% of the participants used health services. In addition, more than 90% of the participants took physical exercise, which indicates that most participants realized the importance of physical exercise.

**Table 2:** Descriptive statistics

Variable	Full sample (*N* = 1,924)	With URRBMI (*N* = 1,593)	Without URRBMI (*N* = 331)
IADL disability			
No^a^, n (%)	1,495 (77.70)	1,267 (79.54)	228 (68.88)
Yes, n (%)	429 (22.30)	326 (20.46)	103 (31.12)
Age			
45–64, n (%)	1,235 (64.19)	1,033 (64.85)	202 (61.03)
≥ 65, n (%)	689 (35.81)	560 (35.15)	129 (38.97)
Gender			
Female^a^, n (%)	1,015 (52.75)	836 (52.48)	179 (54.08)
Male, n (%)	909 (47.25)	757 (47.52)	152 (45.92)
Marital status			
Single, divorced or widowed^a^, n (%)	257 (13.36)	190 (11.93)	67 (20.24)
Married, n (%)	1,667 (86.64)	1,403 (88.07)	264 (79.76)
Residency area			
Rural areas^a^, n (%)	1,187 (61.69)	973 (61.08)	214 (64.65)
Urban areas, n (%)	737 (38.31)	620 (38.92)	117 (35.35)
Education status			
Literate^a^, n (%)	370 (19.23)	289 (18.14)	81 (24.47)
Illiterate, n (%)	1,554 (80.77)	1,304 (81.86)	250 (75.53)
Household income (RMB)			
Mean (SD)	27,789.56 (46,934.82)	29,091.85 (49,294.55)	21,522.03 (32,657.31)
PM_2.5_ concentrations (μg/m^3^)			
Mean (SD)	34.37 (14.65)	34.89 (14.98)	31.87 (12.68)
Having tap water			
No^a^, n (%)	334 (17.36)	271 (17.01)	63 (19.03)
Yes, n (%)	1,590 (82.64)	1,322 (82.99)	268 (80.97)
Health services utilization			
No^a^, n (%)	1,428 (74.22)	1,152 (72.32)	276 (83.38)
Yes, n (%)	496 (25.78)	441 (27.68)	55 (16.62)
Physical exercise			
No^a^, n (%)	184 (9.56)	146 (9.17)	38 (11.48)
Yes, n (%)	1,740 (90.44)	1,447 (90.83)	293 (88.52)

URRBMI indicates Urban and Rural Resident Basic Medical Insurance. IADL indicates instrumental activity of daily living. ^a^ indicates the reference group. RMB indicates Renminbi. SD indicates standard deviation. PM_2.5_ indicates Particulate Matter 2.5

### Regression results of the association between URRBMI and IADL disability

Table 3 displays the logit regression results of the association between URRBMI and IADL disability among the middle-aged and older adults. Models 1–4 reveal that URRBMI participation was significantly related to reduced odds of suffering from IADL disability (*p* < 0.01).

**Table 3:** Logit regression results of the association between Urban and Rural Resident Basic Medical Insurance and instrumental activity of daily living disability among the middle-aged and older adults in China

Variable	Model 1	Model 2	Model 3	Model 4
URRBMI	0.5696^**^	0.5583^**^	0.6039^**^	0.6014^**^
	(0.0845)	(0.0920)	(0.1008)	(0.1034)
Age		1.0660^**^	1.0605^**^	1.0631^**^
		(0.0076)	(0.0074)	(0.0071)
Gender		0.4834^**^	0.5555^**^	0.5557^**^
		(0.0555)	(0.0662)	(0.0673)
Marital status		1.1825	1.2423	1.2905
		(0.1837)	(0.1980)	(0.2097)
Residency area		0.7456	0.7913	0.8927
		(0.1348)	(0.1460)	(0.1577)
Education status			0.6136^**^	0.6260^**^
			(0.1031)	(0.1044)
Household income			0.9270^**^	0.9320^**^
			(0.0177)	(0.0171)
PM_2.5_ concentrations				0.9987
				(0.0069)
Having tap water				0.4713^**^
				(0.0903)
Constant	0.4518^**^	0.0113^**^	0.0354^**^	0.0507^**^
	(0.0533)	(0.0057)	(0.0188)	(0.0310)
Number of observations	1,924	1,924	1,924	1,924
Wald chi-squared	14.39^**^	117.26^**^	152.29^**^	208.52^**^
Pseudo R-squared	0.0083	0.0789	0.0934	0.1068

Robust standard errors that clustered at the community level are reported in parentheses. ^**^p < 0.01. URRBMI indicates Urban and Rural Resident Basic Medical Insurance. PM_2.5_ indicates Particulate Matter 2.5

### IV-Probit analysis

In this section, we used the IV-Probit regression model to address the potential endogeneity problem which is caused by reverse causation and omitted variable bias. Table 4 presents the IV-Probit estimation results of the association between URRBMI and IADL disability among the middle-aged and older adults. The first stage regression results reveal that participation rate of URRBMI at the community level was positively associated with URRBMI participation at the 0.01 level. Furthermore, the second stage regression results indicate that URRBMI was significantly correlated with a reduced possibility of suffering from IADL disability (coefficient = -0.3419, *p* < 0.01). Moreover, Wald test of exogeneity indicates that URRBMI was an exogenous variable (*p* > 0.05), not an endogenous variable. This means that the endogeneity problem was not serious for this study.

**Table 4:** IV-Probit estimation results of the association between Urban and Rural Resident Basic Medical Insurance and instrumental activity of daily living disability among the middle-aged and older adults in China

Variable	First stage	Second stage
	URRBMI	IADL disability
	(1)	(2)
Participation rate of URRBMI at the community level	0.9863^**^	
	(0.0210)	
URRBMI		-0.3419^**^
		(0.1180)
Age	0.0006	0.0355^**^
	(0.0007)	(0.0039)
Gender	0.0040	-0.3371^**^
	(0.0122)	(0.0713)
Marital status	0.0157	0.1438
	(0.0183)	(0.1007)
Residency area	-0.0082	-0.0582
	(0.0124)	(0.0724)
Education status	0.0342^*^	-0.2801^**^
	(0.0159)	(0.0838)
Household income	0.0032	-0.0407^**^
	(0.0019)	(0.0109)
PM_2.5_ concentrations	0.0001	-0.0009
	(0.0004)	(0.0023)
Having tap water	0.0055	-0.4436^**^
	(0.0157)	(0.0840)
Constant	-0.0952	-1.7003^**^
	(0.0576)	(0.3176)
Number of observations	1,924	1,924
F test	257.08^**^	
R-squared	0.5473	
Adj R-squared	0.5451	
Wald chi-squared		199.00^**^
Wald test of exogeneity		0.19

Standard errors are reported in parentheses. ^**^p < 0.01, ^*^p < 0.05. URRBMI indicates Urban and Rural Resident Basic Medical Insurance. IADL indicates instrumental activity of daily living. PM_2.5_ indicates Particulate Matter 2.5

### Robustness checks

In this section, we carried out robustness checks on the previous logit regression results. Firstly, we used PSM to conduct a robustness check, and the results were shown in Table 5. The PSM estimation results reveal that URRBMI participation was significantly associated with a reduced possibility of suffering from IADL disability (*p* < 0.01), which suggests that our previous regression results were robust.

**Table 5:** PSM estimation results for the association between Urban and Rural Resident Basic Medical Insurance and instrumental activity of daily living disability among the middle-aged and older adults in China

Matching method	Sample	Treated	Control	ATT	S.E	T-stat
K-nearest neighbor matching	Unmatched	0.2046	0.3112	-0.1065	0.0250	-4.25
	Matched	0.2049	0.2973	-0.0924	0.0313	-2.95^**^
Radius matching	Unmatched	0.2046	0.3112	-0.1065	0.0250	-4.25
	Matched	0.2050	0.2988	-0.0938	0.0295	-3.18^**^
Kernel matching	Unmatched	0.2046	0.3112	-0.1065	0.0250	-4.25
	Matched	0.2049	0.2909	-0.0860	0.0288	-2.99^**^
Nearest-neighbor matching within caliper	Unmatched	0.2046	0.3112	-0.1065	0.0250	-4.25
	Matched	0.2050	0.2964	-0.0913	0.0313	-2.92^**^

^**^p < 0.01

Secondly, we employed the generalized random forest model to conduct a robustness check. Table 6 presents the generalized random forest model estimation results of the association between URRBMI and IADL disability among the middle-aged and older adults. Models 1–4 reveal that the values of average treatment effect (from -0.1238 to -0.1086) and standard errors (from 0.0310 to 0.0358) barely changed, and the significance level remained unchanged (*p* < 0.01). In addition, the estimation results of the generalized random forest model indicate that the middle-aged and older adults who were covered by URRBMI had a reduced possibility of suffering from IADL disability when compared with those without URRBMI (*p* < 0.01), which indicates that our previous regression results were robust.

**Table 6:** Generalized random forest model estimation results of the association between Urban and Rural Resident Basic Medical Insurance and instrumental activity of daily living disability among the middle-aged and older adults in China

Variable	Model 1	Model 2	Model 3	Model 4
URRBMI	-0.1111^**^	-0.1238^**^	-0.1086^**^	-0.1102^**^
	(0.0336)	(0.0358)	(0.0314)	(0.0310)
Cluster	No	No	No	Yes
Number of decision trees	500	1,000	2,000	2,000
Number of observations	1,924	1,924	1,924	1,924

Standard errors are reported in parentheses. ^**^p < 0.01. URRBMI indicates Urban and Rural Resident Basic Medical Insurance

### Heterogeneity analysis

In order to explore whether the association between URRBMI and IADL disability differs by age and residency area, we used logit regression models to conduct the following heterogeneity analysis.

Table 7 displays the heterogeneity analysis of the association between URRBMI and IADL disability among the middle-aged and older adults. We divided the sample into two age groups, the middle-aged adults (aged 45–59 years) and the older adults (aged 60 years and older). The results of heterogeneity analysis reveal that URRBMI produced a statistically stronger effect on IADL disability for the older adults (OR = 0.5815, *p* < 0.01) when compared with the middle-aged adults (OR = 0.5690, *p* < 0.05). In addition, the results of heterogeneity analysis also reveal that the association between URRBMI and IADL disability was not heterogeneous across residency area (*p* < 0.05).

**Table 7:** Heterogeneity analysis of the association between Urban and Rural Resident Basic Medical Insurance and instrumental activity of daily living disability among the middle-aged and older adults in China

Variable	Model 1	Model 2	Model 3	Model 4
	Middle-aged adults	Older adults	Rural areas	Urban areas
URRBMI	0.5690^*^	0.5815^**^	0.6392^*^	0.5412^*^
	(0.1520)	(0.1205)	(0.1401)	(0.1415)
Age	1.1055^**^	1.0535^**^	1.0749^**^	1.0437^**^
	(0.0312)	(0.0121)	(0.0092)	(0.0116)
Gender	0.6157^*^	0.5220^**^	0.5616^**^	0.5184^**^
	(0.1172)	(0.0878)	(0.0904)	(0.0949)
Marital status	1.1689	1.2838	1.4294	1.1440
	(0.5090)	(0.2324)	(0.2954)	(0.3069)
Residency area	1.2297	0.7399		
	(0.3084)	(0.1433)		
Education status	0.5812^*^	0.6549^*^	0.6843^*^	0.5024^*^
	(0.1544)	(0.1207)	(0.1272)	(0.1689)
Household income	0.9439^**^	0.9201^**^	0.9208^**^	0.9491^*^
	(0.0206)	(0.0257)	(0.0233)	(0.0247)
PM_2.5_ concentrations	0.9789^*^	1.0101	0.9993	0.9985
	(0.0090)	(0.0071)	(0.0086)	(0.0110)
Having tap water	0.3490^**^	0.5916^**^	0.4494^**^	0.5205^*^
	(0.0964)	(0.1156)	(0.1038)	(0.1710)
Constant	0.0139^**^	0.0654^**^	0.0228^**^	0.1683
	(0.0224)	(0.0619)	(0.0188)	(0.1686)
Number of observations	890	1034	1187	737
Wald chi-squared	61.40^**^	81.06^**^	139.32^**^	85.19^**^
Pseudo R-squared	0.0970	0.0817	0.1183	0.0880

Robust standard errors that clustered at the community level are reported in parentheses. ^**^p < 0.01, ^*^p < 0.05. URRBMI indicates Urban and Rural Resident Basic Medical Insurance. PM_2.5_ indicates Particulate Matter 2.5

### Impact channel analysis

To verify the above-mentioned hypotheses, we took health services utilization and physical exercise as channel variables, and then conducted impact channel analysis for the association between URRBMI and IADL disability among the middle-aged and older adults.

Table 8 shows the impact channel analysis results for the association between URRBMI and IADL disability among the middle-aged and older adults. Model 1 reports the main results from Table 3, and Models 2 and 3 controlled the channel variables one at a time. Models 2 and 3 show that URRBMI participation was significantly correlated with reduced possibility of suffering from IADL disability after controlling health services utilization and physical exercise, respectively (*p* < 0.01). The impact channel analysis results reveal that the absolute value of coefficient of URRBMI increased by 21.05% after controlling health services utilization, which indicates that health services utilization was not a suitable channel variable involving the effect of URRBMI on IADL disability. Furthermore, it is found that physical exercise accounted for 4.90% of the association between URRBMI and IADL disability, which suggests that physical exercise was a channel involving the effect of URRBMI on IADL disability.

**Table 8:** Impact channel analysis results for the association between Urban and Rural Resident Basic Medical Insurance and instrumental activity of daily living disability among the middle-aged and older adults in China

Variable	Model 1	Model 2	Model 3
URRBMI	-0.0898^**^	-0.1087^**^	-0.0854^**^
	(0.0291)	(0.0262)	(0.0291)
Age	0.0099^**^	0.0094^**^	0.0091^**^
	(0.0011)	(0.0011)	(0.0011)
Gender	-0.0881^**^	-0.0798^**^	-0.0874^**^
	(0.0185)	(0.0185)	(0.0179)
Marital status	0.0337	0.0291	0.0310
	(0.0285)	(0.0303)	(0.0280)
Residency area	-0.0146	-0.0187	-0.0123
	(0.0260)	(0.0188)	(0.0255)
Education status	-0.0958^**^	-0.1025^**^	-0.0958^**^
	(0.0331)	(0.0282)	(0.0326)
Household income	-0.0110^**^	-0.0104^**^	-0.0101^**^
	(0.0032)	(0.0029)	(0.0030)
PM_2.5_ concentrations	-0.0002	0.0002	-0.0004
	(0.0011)	(0.0007)	(0.0010)
Having tap water	-0.1304^**^	-0.1273^**^	-0.1327^**^
	(0.0365)	(0.0271)	(0.0356)
Health services utilization		0.1638^**^	
		(0.0225)	
Physical exercise			-0.1802^**^
			(0.0309)
Constant	0.0011	-0.0093	0.2112
	(0.1037)	(0.0938)	(0.1093)
1 − *β*/*α*		-0.2105	0.0490
Number of observations	1,924	1,924	1,924
F test	26.88^**^	29.97^**^	28.40^**^
R-squared	0.1138	0.1428	0.1296

Robust standard errors that clustered at the community level are reported in parentheses. ^**^p < 0.01. URRBMI indicates Urban and Rural Resident Basic Medical Insurance. PM_2.5_ indicates Particulate Matter 2.5

## Discussion

Using the data which were obtained from the 2018 wave of CHARLS, we examined the association between URRBMI and IADL disability among the middle-aged and older adults in China. The logit regression results reveal that the middle-aged and older adults who were covered by URRBMI had reduced odds of suffering from IADL disability when compared with those who were not covered by URRBMI. Furthermore, the IV-Probit estimation results indicate that URRBMI was an exogenous variable and the endogeneity problem was not serious. Moreover, the robustness checks using PSM and the generalized random forest model reported similar estimation results. This finding was consistent with Cheng et al. [17] and Zhang et al. [18], who found that NRCMS significantly reduced the possibility of suffering from ADL disability.

We conducted heterogeneity analysis of the association between URRBMI and IADL disability among the middle-aged and older adults in China. The results of heterogeneity analysis reveal that URRBMI produced a statistically stronger effect on IADL disability for the older adults when compared with the middle-aged adults. In addition, the results of heterogeneity analysis also reveal that the association between URRBMI and IADL disability was not heterogeneous across residency area.

We also explored the impact channels for the association between URRBMI and IADL disability among the middle-aged and older adults in China. We obtain evidence indicating that health services utilization was not a suitable channel variable involving the effect of URRBMI on IADL disability. Furthermore, we also obtain evidence indicating that physical exercise was a channel involving the effect of URRBMI on IADL disability. That is to say, health behavior played an important role in explaining the association between URRBMI and IADL disability. This finding was consistent with Fan et al. [50], who discovered that health behavior was a channel involving the effect of public health insurance on health outcomes.

This study has several policy implications. Firstly, it is necessary to integrate health consultation services in URRBMI to strengthen the guidance on physical exercise. Secondly, the Chinese government needs to strengthen health education and publicity, use plain words and develop more short videos to popularize health knowledge, improve the pertinence of health education and publicity, guide the middle-aged and older adults to make health investment in advance, and change unhealthy behaviors, such as controlling smoking, to promote health. Thirdly, since this study finds that about 22% of the middle-aged and older adults suffered from IADL disability, the Chinese government should adjust long-term care insurance to provide better care protection for them.

To the best of our knowledge, this is the first study to explore the association between URRBMI and IADL disability among the Chinese middle-aged and older adults using a nationally representative population survey. In addition, it must be acknowledged that this study does suffer from several limitations. Firstly, considering the fact that the data used in this study is cross-sectional, we cannot fix the endogeneity problem concerning time-invariant omitted variables. Future studies may consider using panel data to explore the association between URRBMI and IADL disability among the middle-aged and older adults. Secondly, due to the short period of implementation of URRBMI in China, this study cannot explore the long-term effect of URRBMI on IADL disability among the middle-aged and older adults. Future studies may consider paying attention to the long-term effect of URRBMI on IADL disability among the middle-aged and older adults. Thirdly, though we have tried our best to control the factors that may be associated with IADL disability, we cannot control some factors, such as health literacy [51–54], due to the limitation of data. Future studies may consider controlling health literacy when data is available.

## Conclusion

In conclusion, this study finds that the middle-aged and older adults who were covered by URRBMI had a reduced possibility of suffering from IADL disability when compared with those without URRBMI. Furthermore, it is found that URRBMI produced a statistically stronger effect on IADL disability for the older adults when compared with the middle-aged adults. Moreover, we obtain evidence indicating that physical exercise was a channel involving the effect of URRBMI on IADL disability.

## Data Availability

The data used in this study are drawn from the China Health and Retirement Longitudinal Study.
